# Exploring the Processes Involved in Seeking Help from a General Practitioner for Young People Who Have Been at Risk of Suicide

**DOI:** 10.3390/ijerph18042120

**Published:** 2021-02-22

**Authors:** Jack Farr, Andrew D. R. Surtees, Hollie Richardson, Maria Michail

**Affiliations:** 1 Centre of Applied Psychology, University of Birmingham, Birmingham B15 2TT, UK; hxr227@alumni.bham.ac.uk; 2 Birmingham and Solihull Mental Health NHS Foundation Trust, Birmingham B1 3RB, UK; 3 Centre of Applied Psychology, School of Psychology, University of Birmingham, Birmingham B15 2TT, UK; A.Surtees@bham.ac.uk; 4 Birmingham Women’s and Children’s NHS Foundation Trust, Birmingham B4 6NH, UK; 5 Institute for Mental Health, School of Psychology, University of Birmingham, Birmingham B15 2TT, UK; M.Michail@bham.ac.uk

**Keywords:** help seeking, suicide, primary care

## Abstract

General practitioners (GPs) play a key role in the early identification and management of suicide risk in young people. However, little is known about the processes involved in how, when and why a young person decides to seek help from their GP. Eight young people, aged 17–23, took part in semi-structured interviews exploring their experiences of help seeking when feeling suicidal. Data were analysed using framework analysis. The analysis identified three main themes and seven subthemes. The main themes explored were: understanding when to seek help from a GP, barriers and facilitating factors at the GP consultation, and help seeking as a non-linear and dynamic process. The processes involved in how, when and why young people seek help from a GP when feeling suicidal were found to be dynamic and to fluctuate over time. Help seeking was initially related to how young people were able to understand and articulate their distress, the availability of informal support networks, and their perception of the GP as a source of help. During a GP consultation, help seeking was influenced by how safe and supported the young people felt. Perceived GP training, communication and validation of young people’s concerns were important factors to help facilitate this process. Subsequent help seeking was influenced by prior experience of GP consultations and the availability of alternative support.

## 1. Introduction

### 1.1. Suicide in Young People

Suicide is a complex public health problem that can affect individuals, families and wider communities [1]. Suicide is a global problem, and was recorded as the second leading cause of death among 15 to 29 year olds worldwide, accounting for 8% of all deaths [2]. Many countries have seen a sustained increase in youth suicide rates in recent years—greater than in any other age group [3]. In the UK, in 2018, there was a 22% 1-year increase in suicide rates in those under the age of 24 and the highest ever recorded suicide rate for young females since 1981 [4]. The World Health Organisation (WHO) estimates that for each person who dies by suicide, there are a further 20 people who have made a suicide attempt [5]. Suicide attempts, suicidal ideation and self-harm are also very high in this age group and are associated with completed suicide [6]. Self-harm in particular has been shown to be the single greatest predictor of suicide, with 50% of young people who died by suicide having previously self-harmed [7].

### 1.2. Preventing Youth Suicide: The Role of General Practitioners

Given the size and scale of youth suicide and the impact that suicide can have on individuals, families and communities, the reduction in suicide among young people has been highlighted as a global health target [5]. Indeed, the reduction in deaths by suicide is a priority for the WHO in their mental health action plan (2013–2020) and it has also been highlighted as an indicator of progress in the United Nations Sustainable Development Goals [8]. To achieve this target, a comprehensive and coordinated response to suicide prevention is needed where the early identification and management of high-risk groups, such as young people, is key.

We have previously argued [9] that the role of primary care, within a multifaceted public health approach to suicide prevention, is vital, with evidence showing increased rates of contact with their GP among young people who have completed suicide one to three months prior to their death [10,11]. The increasing frequency of GP consultations has been associated with rising suicide risk, with the highest risk among those who consulted their GP over 24 times during a 12 month period [12]. Approximately 40% of all GP appointments have a mental health element, with GPs often fulfilling the role of a ‘gatekeeper’ to specialist mental health support [13]. GPs are therefore in a unique position to identify early and support young people at risk of suicide.

Whilst the GP consultation provides an excellent opportunity to support young people at risk of suicide, there are many challenges that GPs can face. For example, time constraints, heavy workload, lack of integration with mental health services and a lack of specialist education has left many GPs feeling not adequately prepared to meet young people’s complex needs in relation to suicide risk [9,14,15].

### 1.3. Young People’s Perceptions of the GP’s Role

Although the role of GPs as a source of support for mental distress has been identified as important [9,10,11], evidence on young people’s views around GPs as a source of help seeking for mental health and suicide-related experiences is mixed [16,17,18]. There is some evidence [19] showing that GPs are a trusted source of formal help seeking, suggesting that young people may move through a process of recognition, identification of the GP as a source of help and then subsequent willingness to disclose risk. However, other evidence [16] suggests that young people can fall into a cycle of avoidance that seeks to normalise how they are feeling through their life experiences in an attempt to avoid acknowledging that they are in real distress. It is possible therefore that young people can become ambivalent about seeking help from their GP and research suggests that young people may prefer to seek help from informal sources such as from their peers [20,21,22]. Indeed, Biddle and colleagues [16], exploring young adults’ perceptions of GPs as a source of support for mental distress, found that young people did not value or recognise that the GP could offer support for mental health difficulties; rather, that their role was limited to provision of help for physical health problems. Young people also thought that the most likely outcome from attending a GP consultation would be a prescription of antidepressants rather than provision of ‘talking therapies’. Understanding the processes underlying young people’s help seeking from a GP when feeling suicidal is vital both to help improve their experience of seeking and accessing support from primary care but also to inform future conceptualisations of help seeking.

### 1.4. Research Aims

This study aims to explore how, when and why young people seek support from their GP when they are feeling suicidal. By gaining in-depth knowledge of the processes that young people employ in regards to seeking help for suicide experiences, future educational interventions for GPs may be better tailored to young people’s experience and needs. Furthermore, additional support can be put in place to improve access for young people to receive appropriate care that takes into account their views and experiences of seeking help for suicidal ideation.

## 2. Materials and Methods

### 2.1. Ethical Approval

Ethical approval for this project was granted from the National Health Service’s Health Research Authority (NHS HRA, REC reference: 19/WM/0104). Ethical considerations around consent, confidentiality and anonymity were taken into account, as well as ensuring that participants felt that they were safe to participate, and that participation did not cause distress.

### 2.2. Study Design and Setting

This is a qualitative study using semi-structured interviews with young people who were under the care of a children, young people and families mental health service in the UK. The service provides mental health support across four community hubs and one inpatient setting; however, for the purposes of this study, only the four community hubs were approached. The local population is diverse and populous. The use of a semi-structured interview ensured that relevant topics were covered, but it also enabled the collection of flexible and open responses from the participants [23]. This study is conducted in line with the consolidated criteria for reporting qualitative research (COREQ) [24].

### 2.3. Sample and Recruitment

The current study used a convenience sampling method relying on volunteers. Multidisciplinary team meetings were attended at local children, young people and families service hubs and care coordinators were approached directly about the research to inform them of the study eligibility criteria. Care coordinators then approached young people on their case load, giving information about the research and seeking consent to be contacted by the researcher if they expressed an interest in taking part. The researcher also met with and advertised the research through the service’s Youth Advisory Group; on social media; and a snowball sampling strategy was also employed to encourage further participation in the research.

Inclusion criteria for participation were as follows:

- Aged 16–25 years old.
- History of suicide attempts in the past three years (excluding a period of six months before the interview).
- Participants must be registered with a GP in the local area and be under the care of the local children, young people and families mental health service.
- Participants must be able to provide informed consent.
- Participants must have sufficient command of English.
- No restriction placed on current diagnosis because suicide is a transdiagnostic experience.

In total, eight participants were recruited and took part in the semi-structured interviews and no participants withdrew from this study. All participants were given a pseudonym to protect confidentiality. Six participants identified as female and two participants identified as male. Participants were aged 17 to 23 years old and had received various mental health diagnoses such as bipolar disorder, depression, and anxiety. No further information was collected about the participants as it was not necessary for the present study.

### 2.4. Material

The semi-structured interview (Appendix A) was developed by researching the existing evidence base around young people, help seeking and primary care; and subsequently reviewed by the research team. The refined interview schedule was then sent to a Youth Advisory Group of young people with lived experience of mental ill health in order to seek their views on the acceptability of the topic guide and to ensure the questions were meaningful to them. No changes were made by the youth advisory group.

### 2.5. Data Collection

Each participant took part in a semi-structured interview that lasted approximately one hour, with a follow-up debrief session offered with a Clinical Psychologist. However, none of the participants took up the debrief session that was offered. A flexible schedule of open-ended questions was used to guide the interviews, which allowed for further exploration, clarification and probing where appropriate. All interviews took place at the young person’s local children, young people and families mental health service hub. Each interview was conducted, audio recorded, transcribed verbatim and checked for accuracy by the lead author (JF). Participants were made aware that they could withdraw from the research before, during or after data collection. All participants chose to be interviewed alone and there were no repeat interviews.

### 2.6. Data Analysis

Data were analysed using a thematic framework method [25]. The framework method is an approach to qualitative data analysis that allows themes to be derived from the narratives of the participants as well as from the research question [26]. The framework is a pragmatic approach to analysis and is increasingly common in health care research settings [27]. Framework analysis was chosen as the present research aimed to illicit the participant’s ‘story’ of seeking help, taking into account context, experience and process. Framework analysis has been highlighted as being a suitable approach for this type of research as it emphasises how both a priori issues and emergent data-driven themes guide the development of the analytic framework [28].

Framework analysis uses several inter-related distinct stages [29] that allow for both theme-based and case-based analysis utilising the development of a framework matrix. One of the biggest strengths of framework analysis is that it is a straightforward and transparent process with clear stages [30] that also provides a clear audit trail of the analytical process [31]. The present research followed the stages described within the ‘illustrative example of the use of the framework method’ [32]. This method is not aligned with a particular epistemological, philosophical, or theoretical approach. Rather it is a flexible tool that can be adapted for use with many qualitative approaches that aim to generate themes [32].

Interviews were transcribed verbatim by the lead author (JF). The author then coded the first two transcripts, highlighting important segments of text and applying a label or code along with more detailed ideas and notes (Figure S1). A second researcher (HR) then also followed this process independently from the lead author (JF). The two researchers (JF and HR) then met to discuss the codes and labels which were then sorted and the first analytical framework was developed (Figure S2). This framework was then applied to each of the transcripts, reviewing and summarising the framework whilst making connections both within and between participants (Figures S3 and S4). Ideas were explored in consultation with the second researcher (HR) and reviewed by the last author (MM). A summary of the results was returned to participants with the possibility for them to make comments or corrections.

To establish methodological rigor [33], the data analysis followed a systematic process with a clear audit trail. Researcher triangulation was employed with discussion and refinement of the thematic framework to ensure there was consistency and accuracy between the integration of data and interpretations. The findings were discussed in consultation with other researchers and sent to participants to improve credibility as well as quotes provided as examples of particular themes [34]. Results from the present research have then been compared with existing literature to confirm and expand upon this study’s findings.

## 3. Results

Three main themes and seven subthemes were identified from the interviews. The overarching themes cover the process of help seeking from a GP; starting with the process of when young people first considered seeking help, the process when they tried to seek help, and how the process changed following their GP consultations. All participants contributed to each theme. However, any commonalities or variations within each theme have been highlighted in the text.

### 3.1. Understanding When to Seek Help from a GP

All participants had attended a consultation with their GP that involved discussing their suicidal experience. In understanding how the participants came to attend their first GP appointment, three subthemes were identified: difficulty with understanding and articulating internal distress; arranging and accessing a GP consultation; and preconceptions of the GP’s role. The three subthemes are outlined below.

#### 3.1.1. Difficulty with Understanding and Articulating Internal Distress

For all eight of the young people interviewed, the development of their suicidal experience was described to have started in their late childhood years and early teens. Young people attributed their developing difficulties to various levels of adverse childhood experiences. For example, participants described experiencing verbal and physical abuse as well as difficulties with school transitions, bullying and relationship or family breakdowns.

However, although the young people were explicit in their descriptions of what was happening to them during these years, there was a general confusion or difficulty in understanding and making sense of their internal emotional state. Participants were aware that something was not quite right in how they felt but did not feel that they had the words to describe their experience to others.

“*I was a kid, I was scared. I didn’t know what the hell was going on. My childlike instinct was screaming inside of me ‘this ain’t right, you should be scared.’ I guess deep down, below my mental health demons, deep down, I really did want to be helped, and I wanted that support.*”

(Sophia)

“*To me, it was almost like an overflowing cup. A cup, which kept getting full, filling up, and then there was just one night and – Again, I can’t actually say what happened, it was just too much*”

(Sarah)

In the quote above, Sarah uses the metaphor of a cup that was steadily filling up, and alludes to how in one night it overflowed. In this way, we can get a sense of what may have happened that night, even though she did not describe the events. This illustrates how difficult it is to put into words the experience of the suicide attempt and yet still conveys some of the meaning. Furthermore, the quote by Sophia highlights that even when it is difficult to make sense of how she was feeling, she still wanted help. Sophia described this period of her life to be scary and how the idea of help seeking was supressed by what she described as her ‘mental health demons.’ The description presented here perhaps further highlights what a scary experience this was for a child and also some of the complexities young people face when trying to describe or explain how they are feeling.

Given some of the difficulties in finding the right words to explain their internal experience, participants often used physical symptoms to try and make sense of and explain what was happening to them. However, it was difficult to link this experience to their developing suicidal feelings or mental health difficulties.

“*I just had this horrific anxiety, but I didn’t know what it was. I didn’t really understand mental health as a concept. Then I described to my friends that before we were going to go out, I just had this feeling of being sick or not being able to go*”

(Olivia)

Participants used different language and ways of talking about their difficulties, which is likely influenced by their individual culture, family upbringing and exposure to others with mental health difficulties.

“*They [parents] were very religious and they didn’t understand that much about how they behaved. […] They never really understood why I would cry so much and why I was so sensitive. My parents made me go through two exorcisms and the first was when I was 11, and that was quite traumatic for me.*”

(Prisha)

“*I didn’t understand my mental health in general growing up. I find it hard to talk about things. I know my Dad grew up around it, not necessarily had it himself, but I have got my older sister who he has been looking after […] My sister has got scars all over her arms and she never told me why. Obviously because I was a child at the time, I thought she just cut it on a fence or whatever she says.*”

(Liam)

In the quotes above, Prisha speaks about a significant traumatic experience in her younger years that she believes was linked to her family’s’ lack of understanding of mental health difficulties. She attributed her experience to her family’s religion and how they perhaps believed that Prisha was possessed. Liam, on the other hand, alludes to a family where mental health difficulties were always present; however, this did not mean that they were spoken about openly. As a child, he was protected from understanding what was happening with his family, as far as believing that his sister’s scarred arms were a result of cutting them on a fence. It is possible Liam could not find the words to explain his own feelings because these feelings were not spoken about within his family. These quotes perhaps also further highlight how young people may struggle to understand their internal experience based more broadly on external factors such as family and religion.

As the young people found it challenging to make sense of their difficulties and had trouble finding the words to articulate their internal experience, this had a direct consequence on the process of seeking help. Some participants were very explicit in their worry that they would be dismissed because they could not clearly describe how they were feeling.

“*I think there is such a depth to my struggles that I didn’t quite have the words to explain. I didn’t want to be turned away because I was explaining it in a light-hearted manner*”

(Hayley)

Hayley summarises this subtheme in the above quote. She did not believe she had the words to be able to articulate the severity of how she was feeling. She speaks about describing her struggles in a ‘light-hearted’ way and as a result is concerned about not being understood or taken seriously. In Hayley’s example, it is clear that her ability and confidence in describing her distress was preventing her from seeking help at this point in her life.

#### 3.1.2. Arranging and Accessing a GP Consultation

Among those participants that had a supportive network, there was a general consensus that it was their support network that first identified that something was wrong and that help was needed. Some families and friends were able to draw on their knowledge or experience of mental health difficulties or their own life experiences to understand what might be happening. However, for others, people in their support network were as confused as the young person. Sometimes families did not know what to do and felt that the GP was the natural contact that would help make sense of the presenting difficulty.

“*My mum was concerned because she didn’t know what was going on, we had been the perfect family. She didn’t understand what was going on because I was the oldest child in the family, we hadn’t had any run-ins with mental health thus far, and so it was scary for her. I think she was hoping that it (contacting the GP) would change things and that I would get better. She was concerned and she felt out of her depth I guess.*”

(Sophia)

In the quote above, Sophia illustrates how mental health difficulties can present even in a ‘perfect family.’ She alludes to how scary this situation can be, not only for the young person, but also the family. For Sophia, it felt that the process of consulting a GP was driven by her mum, in a desperate attempt to help Sophia get better. The quote further suggests a sense of apprehension that can underlay the first contact with a GP.

Seven out of the eight participants described being taken to the GP by either their family or friends at least once. At the first consultation, there was a mix of feelings around this for the young people. One participant described feeling hopeful that something might change and that they may begin to feel better.

“*People are not just made better by nothing, you have to actually do something. I remember before going to the GP, I kept asking my mum if we could go to the pharmacy, to see if we could buy something to help with my anxiety or help with my mood. My mum took me straight to the GP*”

(Connor)

However, for other young people, there was a worry that they were not ready to open up about how they were feeling; or they were worried that by disclosing to their GP what was happening, it would take away the legitimacy of their suicidal experience. There was a sense that the internal struggle that young people were battling with would be minimised, not taken seriously or even perceived to be seeking attention.

“*I was not ready to deal with anything, I didn’t want to talk to the GP and go through everything, but when I was younger, my parents just carted me up to the GP*”

(Sarah)

“*If you keep it to yourself, it remains a valid attempt, a valid experience.” […] “It is such a serious decision to make [to act on suicidal feelings], that if you try and seek help and you get like a ‘you are doing this on purpose for a different reason, or for attention’ it makes a mockery of what you were feeling*”

(Olivia)

In the quote above, Sarah is talking about being forced by her parents to see the GP despite her worry and concerns that she was not ready to talk about her difficulties. In the second quote by Olivia, she is talking perhaps more about the idea that if she did attend the GP consultation, this would compromise, in her eyes and those of others, the validity of her suicide attempt. In her words, it is only a ‘valid’ suicide attempt if you ‘keep it to yourself’.

For participants who did not feel that they had a supportive network, one participant went to the GP alone, whilst others had experience of bypassing the GP and going straight to A&E where they were taken into psychiatric inpatient units. Two participants described police involvement when it became apparent that they were actively suicidal. Based on young people’s accounts, it appears that they did not have a sense of when they should access the GP and often intervention by others was required to ensure that they received proactive support.

#### 3.1.3. Preconceptions of the GP’s Role

One of the most important factors that deterred young people from seeking help from their GP was the perception that the GP could not offer any help. For many of the young people, they could not understand what was happening to them, others around them could not make sense of it either, so there was little hope a GP would be able to offer any support. It was often the case that participants saw GPs as dealing only with physical health difficulties and as such they could not understand why they needed to access a GP for support.

“*I remember sitting in the waiting room, and that was not pleasant because people were coughing and sneezing, and I was like ‘why am I here? I am not coughing or sneezing’ I had lots of internalised stigma about the GP, without really knowing it”*

(Hayley)

Hayley talks about her experience of sitting in the GP waiting area, feeling confused why she was there and comparing herself to people who are physically unwell. She suggests that it was only at a later date that she realised she had such a strong preconception around the role of the GP. However, at the point when she needed help around her mental health difficulties, she did not see a role for the GP. This view appears more based on Hayley’s understanding on the GP’s role within a mental health context, rather than viewing the GP as not competent.

Other participants felt that the GP might not be able to help, but they saw them as a gatekeeper to better support. There was a hope that by talking with a GP, the young people would be either given medication or therapy to help with their difficulties.

“*They filter out who needs the care. It is a cruel process, but I feel they are the first point of call for support. They are a bouncer to the mental health club*”

(Sophia)

Sophia makes the comparison to entering a club and how you have to get past the bouncer before you enter. She describes the process as cruel, alluding to the idea that some people will go without support; in the same way, a bouncer would reject some individuals from the club. However, she also suggests this to be necessary to get the right support and if we took the metaphor further, it perhaps is about ensuring that the club does not reach capacity. This subtheme highlights how some participants showed a reluctance to approach a GP and have to discuss their difficulties, whilst others show almost an acceptance, seeing the GP as a hoop to jump through to better support.

### 3.2. Barriers and Facilitating Factors at the GP Consultation

All participants at the point of interview had experienced more than one GP consultation, many with different GPs. One participant had entirely positive experiences with their GP, whilst most participants had mixed experiences. Throughout the interviews, it was clear that prior experience of GP consultations was one of the most important factors that would influence whether young people would consider the GP in their process of seeking help in the future. The subthemes relating to facilitating factors and barriers at the GP consultation are detailed below.

#### 3.2.1. Facilitating Factors to Help Seeking at the GP Consultation

Participants spoke about the importance of the GP’s approach in enabling them to feel safe enough to open up about their difficulties. Overwhelmingly, participants found that if they were met with a GP who was welcoming, attentive and made an effort to build up a rapport, then the young person felt more able to disclose how they were feeling and hopeful that they would receive good care.

“*She took care of me because she was talking to my mum and me about how we were both feeling about it. She was very attentive and although she wasn’t my main doctor, she is one of my favourites personally because I just feel really looked after whenever I go to see her*”

(Sophia)

“*The other Dr I saw the year before, who was immediately like ‘come in,’ and then made eye contact with me. That made a huge difference. The subtleties of body language can really tell you a lot about someone’s empathy for you when you are having a mental health crisis*”

(Mia)

Sophia describes that even when a GP is unfamiliar to you, a warm and caring approach can go a long way. In her quote, she talks about how attentive the GP was to both her needs and the needs of her mother. This is very important as we established that many participants attended their GP with a parent or friend. Mia further highlights the importance of a GP’s communication skills, particularly non-verbal, during a consultation. She speaks about the GP ‘immediately’ speaking with her and making eye contact and this approach helped her feel like the GP would be able to empathise with her. We get the sense that the GP had created an open and safe space in which young people could openly talk about their mental health difficulties and feel listened to.

Participants felt that it is important that the GP takes what they are saying at face value and will adapt to how the young person is presenting. Some participants felt that if they are able to talk and explain themselves, they should be given that chance to do so. However, sometimes the young person might feel unable to find the words or are too emotionally suppressed that they do not feel able to talk. In these cases, it was helpful for the GP to take a more direct approach to their assessment.

“*It is important for the GP to be an active listener, being open, and looking like they are interested in the human that is in front of them, because you are human. It may not feel like it at the time, but you are. The GP needs to take charge when they need to, not when they want to” […] “ I think if someone is ‘able’ like in my first appointment, if they can engage in conversation with the GP about how they feel, they need to go with what they are saying. Get what they need from them, with what they are saying. If someone is not able to talk and they are quiet, then ask direct questions. They need to use their training*”

(Hayley)

Most participants found it easier to talk about and disclose information relating to their suicidal experience after getting to know their GP. The young people that were interviewed felt that it took a lot of bravery to talk about their difficulties to a GP and they had to feel safe to be able to do this. It sometimes took more than one GP consultation before the full picture had been disclosed and the right support was offered. It is possible that young people were withholding information based on their experience with their GP, but it is also possible that young people were finding it difficult to know what was important to disclose, or find the right words to convey how deeply affected they were by their internal difficulties. Sophia describes the value of having a good relationship with her GP and she alludes to how it can be helpful to have someone follow your mental health journey from start to end. The positive rapport that they built up together allowed her to feel that she had someone who was rooting for her, and this actually helped with her journey towards recovery.

“*She has been with me from the beginning of the bad times, to coming out of my mental health problems, and it felt really nice to have closure with her” […] “She referred me to CAMHS and got me into that, it made me feel quite proud of myself and everything that I had achieved*”

(Sophia)

All participants spoke about the value of taking family or friends to the GP consultation in regards to their ability to seek help from a GP. Participants found it helpful to have someone available to articulate and explain how they were feeling, when perhaps they could not find the words themselves. Furthermore, participants described a sense that the GP would take the assessment more seriously if the young person had support during the consultation. A few participants also described the value of their GP giving time for the individual to speak alone as well as time to discuss their difficulties, supported by a family a friend.

“*If I had a supportive caregiver around, Mother or Father, somebody else, then I probably would have asked them to come with me, and maybe speak a bit on my behalf. At least it would have been helpful for them to speak initially, and then when I had calmed down, because I used to get very anxious, because of how I had been treated by GP’s, then I would have felt able to chip in with more information for them later on in the consultation*”

(Mia)

“*My mum pushed for my help, which otherwise, if I had gone on my own, downplaying everything, I would have been sent away*”

(Sarah)

The quote by Sarah clearly illustrates a worry that, without her mum, she might not have got the right support. There is a suggestion that the GP might not have understood the difficulties she was experiencing or take it as seriously without a parent present. Mia, who was the only participant who attended her first GP appointment on her own, still spoke about how she would have valued having someone with her to support her to talk about her difficulties. This highlights the significance of other people in the processes of help seeking from a GP.

#### 3.2.2. Barriers to Help Seeking at the GP Consultation

All of the participants spoke about barriers that they experienced at the GP consultation. The 10 minute consultation time came up across by all participants, and it was felt that this was not sufficiently long enough to be able to disclose and talk about their mental health difficulties. Participants did not feel that GPs were well equipped to support their mental health difficulties or assess for suicidal risk. Young people talked about how GPs should have wider training that taught them how to “just be around someone” who was experiencing mental health difficulties, because often they found the GP consultation to be a very invalidating experience.

“*I feel like they need to be taught a process of how to actually be with somebody who is saying all that (disclosing suicidal thoughts), because if you are sitting at your keyboard, not looking at them, part of that training has to be how to sit with and be with that person*”

(Olivia)

“*From my experience with certain GPs, I think they are like the police force. They get a 10-minute lecture on mental health and that is it. But the GPs, I don’t know if they are that interested*”

(Hayley)

“*If they had more training into what to look out for and signs and symptoms that definitely say ‘yes, this person needs to be in services. This person needs extra help.’ I think that would be very helpful.*”

(Sarah)

The three quotes above by Olivia, Hayley and Sarah all talk about their experience or perception that their GPs had not received adequate mental health training. Olivia speaks about a need for general training about how to be with a patient in distress, whilst Sarah talks more about specific training around assessment, diagnosis and management of mental health difficulties. The quote by Hayley raises the question, in her mind, whether GPs are actually interested in mental health—a view shared by other young people in this study who tend to primarily associate the role of the GP with physical health management.

In some cases, the GP had made unhelpful comments or had been dismissive of the young person’s concerns entirely. This fuelled the young person’s suicidal experience by engaging in further self-harming behaviour or even a suicide attempt attributed to a sense of hopelessness following the GP consultation. Mia spoke about how she had felt able to disclose an incident where her ex-boyfriend raped her only for the GP to minimise this experience as a “relationship breakdown”:

“*I think it is very significant, negatively, that she [the GP] attributed my low mood to a ‘relationship breakdown’, which was a complete misunderstanding of what had happened to me*”

(Mia)

Another participant attributed blame to the GP for the increase in his suicidal experience, and felt that it would have been their fault if he had died. This quote shows how a GP’s dismissive comment of the severity of this young person’s symptoms had potentially serious consequences. The dismissal led to the young person feeling that it would have been the GPs fault had they attempted to take their life following the consultation.

“*It would have been their fault [the GP], because it wouldn’t have been my decision to do that [to kill himself]. I understand about not giving me medication because I am complex, but the things that he said like ‘other people are worse off’, he didn’t have to say that at least*”

(Connor)

It was often the case that participants got a sense of whether they would feel able to disclose their feelings and seek help from their GP based on the first few minutes of the consultation.

“*You would get a sense as soon as you walked into the room of whether it was going to be helpful or not. Because, it was how quick they ushered you to the seat, quickly got into their keyboard, typing really fast as you were talking, not even looking at you. You get that sense of nothing is going to happen here*”

(Olivia)

Olivia clearly describes the ‘sense’ you get from the moment you walk into the room for a GP appointment. However, she also went on to speak about how barriers to getting the right support can be internal to the young person. Sophia describes how her suicidal experience was a private experience and she was worried about seeing her family GP, in case the GP disclosed information to her family. Other decisions to withhold information from the GP by other participants were made around not wanting to burden the GP, not wanting to waste the GP’s time or a fear the young person will be labelled as a ‘mental health patient’.

“*I felt really uncomfortable going to the GP, knowing that he has treated me, in theory all my life, and that they knew my mum. I knew that there were professional standards and that they had to have confidentiality, but I thought to myself like ‘being a family GP, I don’t actually trust that the next time he is not going to be like, how is Olivia finding the medication?’ or something that assumed I had told my mum. I always had a funny feeling about that.*”

(Olivia)

In the above quote, Olivia talks about the fear of disclosing information to her GP. There is a sense that perhaps there is some shame, or at least secrecy around her suicidal experience. She alludes to the idea that perhaps it is more painful or worrying to disclose her internal distress to a familiar GP and it would have perhaps been easier to talk to a professional who was unfamiliar to her and her family. This is a contrasting view to other young people who spoke about the value of having a GP who is familiar to them and knows them well. Perhaps the key difference here is that the GP knew Olivia before her suicidal feelings developed, and the GP has a good relationship with her whole family. However, there were general concerns raised that the GP could make assumptions based on what they thought they knew of the young person, leading to them minimising or trivialising their experience. This is clearly illustrated in the quotes below.

“*They asked me ‘are you getting suicidal thoughts? Are you suicidal?’ and when I said ‘yes’. Especially this one Dr, I won’t name names, but he was like ‘you wouldn’t do it anyway’*”

(Liam)

“*If you are sat there explaining you are struggling and you need help, then they should listen and not be like ‘you are just having a bad week’ kind of thing*”

(Sarah)

“*The GP said, when I went into my low mood, he was like ‘You have got a really nice supportive family so you are going to be okay’. I was just thinking ‘you don’t know anything’. He not only made that assumption, but he introduced that concept in the room. I had nowhere to go.*”

(Olivia)

The final quote above by Olivia summarises the danger of making assumptions when assessing risk and the impact this had on her. She talks about how not only did her GP assume that she had a supportive family, she then felt like she perhaps could not challenge this assumption any further, and felt stuck without further support from her GP.

Participants who had experienced barriers at their GP consultation were less likely to open up or seek help from their GP in the future. At times, it made their suicidal feelings a lot worse and their experience reinforced some of their previously held beliefs around GPs not being in a position to offer help, and that nobody could understand or offer support for their internally held difficulties. Some participants felt very uneasy during the consultation and tried to drop ‘hints’ about how they were feeling, or presented with very physical symptoms. It is possible that this approach is seen as the safest route to seeking help, but it relied on the GP picking up these clues, reading the young person’s notes and asking the right questions. It is also possible that if young people view a GP as somebody who only works with physical health difficulties, and if the young person has been taken to see their GP, this could prime them to talk about physical symptoms rather than their emotional experience.

“*I think that if a person doesn’t come in to a GP appointment for their mental health, and they only talk about their physical health, you should still ask about their mental health, because you are a general practitioner. The mind is included in that general bit. I think if someone has pre-existing or pre-diagnosed mental health conditions comes in to your practice with a physical health condition, don’t assume that it is to do with their physical health, because it probably isn’t. Sometimes maybe, but probably not. Just be holistic is my speech of the day.*”

(Hayley)

Hayley summarises this point, and indeed the complexity of the barriers that are at play during a GP consultation. She is speaking about the importance of a holistic assessment as young people may have internally held barriers to disclosing their distress. She suggests that it is the responsibility and part of the role of a GP to ensure that they assess for mental health difficulties, particularly if there is a prior history or evident risk factors. However, as discussed, the internally held barriers that young people hold may also interplay with organisational barriers such as time-limited consultations, as well as internal barriers held by GPs themselves. The interplay of these different barriers presents a significant challenge to the process of seeking help for young people who are feeling suicidal. 

### 3.3. Help Seeking as a Non-Linear and Dynamic Process

Young people referred to the process of help seeking as being a fluid and dynamic process influenced by different factors. Of particular importance were the two subthemes around changing support networks and prior experiences of GP consultations. These subthemes are outlined further below.

#### 3.3.1. Changing Support Networks 

The participants’ support networks (family, friends, peers, etc.) heavily influenced the process of seeking help from a GP. For many participants, after going through the GP consultation and getting into specialist mental health services, they described how the process in which they would now seek help had changed because their support network had also changed. For many, this was a positive experience and they felt better supported than ever before. Nearly all the participants had access to a Community Psychiatric Nurse (CPN) and they spoke about a strong preference to be seen by their CPN, or duty worker, as they preferred to talk to someone who knew them and were ‘mental health trained’.

“*That is what they (CPN) specialise in; it is obviously not just depression. There is a whole wide range of people here that has lots of different mental health problems. I feel because the people that work here are trained in some way in mental health, that is why I feel safer to come here.*”

(Connor)

In the quote above, Connor has a strong preference to speak to someone whom he trusts and who is trained in understanding and supporting people with mental health difficulties. Of particular importance is how Connor describes that knowing that the staff are trained helps him to feel ‘safe’. Indeed, he uses the word ‘safer’, referring to feeling safer than going to a GP. 

Some participants met other young people with mental health difficulties through the services they had gone through, such as inpatient stays, or met partners that they felt more able to disclose their mental health difficulties with. There was an emphasis on how participants had a shared experience with their newly built support network which they perceived to be invaluable.

“*I don’t have many friends anymore because of my mental health. The people that I do have close are through hospital or have got mental health problems themselves. It has taken me a while to find healthy relationships, although they have mental health problems too, they won’t use you and affect my mental health. It has helped a lot to speak to people*”

(Liam)

“*I am very open with him (partner). I tell him everything. He is aware of all my history, all my struggles. It was like having another me, but with more insight.*”

(Sarah)

Liam describes the close relationship he had built up with other young people that have shared experiences with him. He met these people through services that he accessed through his GP. In Liam’s case, it may be that the process of seeking help from a GP will come further down the line as he has a stronger support network in place now. In Sarah’s quote, she describes having a new partner that she feels she can trust and talk openly with about her difficulties. This is a stark contrast to how she had previously held her difficulties to herself and did not feel able to seek help. This is important again for two reasons. Firstly, she has someone she feels is like her, to whom she can talk and use as a source of support. Secondly, she also has someone else who can monitor for risk factors, signs of relapse and someone who can support if another GP consultation is required.

However, some participants spoke about a reliance on the new support that was not always permanent. A lot of services can only provide support for a specified amount of time. Participants found that they can at times become reliant on the support that is put in place and can then relapse when it is taken away.

“*I would get attached to these staff because they would be giving me positive support, and then when I was better, I came out of feeling suicidal. Then it would be like, because I was doing well, it felt like they didn’t care anymore, so then I would be suicidal again. It was a bit of a cycle.*”

(Prisha)

Participants were often reluctant or frustrated about having to go back through the GP. However, after going through services multiple times already, many participants had developed more confidence or assertiveness around telling their support network or GP what they needed.

“*I still rely on people picking up cues. I make them easier for people to find now and when I am feeling more well, I tell people what the cues are so when I am not well, I can use them and know that they already know what they are.*”

(Olivia)

Olivia describes how in her seeking help she heavily relies on other people to pick up the cues, particularly when feeling unwell and perhaps unable to articulate her distress. This further highlights the important role of an informal advocate in supporting young people through their help-seeking pathways.

Young people reported how, in some cases, being assertive with their GP was very helpful, but in other cases this felt like it could develop into conflict when trying to assert what they need with a GP. This confrontation had the potential to cause further reluctance or frustration with the help-seeking process. The quote below illustrates the power imbalance and invalidation of personal experience of mental health difficulties.

“*You try and tell them (the GP) what they have to do, in a way, they don’t like it. They just see it as ‘I have been studying this for ten years, I have qualifications right here.’ Me, personally, I know personal experience is the best qualification for mental health*”

(Liam)

In general, what young people were describing is how they were now drawing on newly built support networks or their greater understanding of their mental health difficulties and this changed the process in how they go about seeking help, if at all, from their GP. Some participants also received various diagnoses across their mental health journey, and found that they were better able to communicate their needs to a GP.

#### 3.3.2. Drawing on Past GP Consultations

Young people’s experience of a GP consultation did not just affect the help-seeking process during that consultation, but it also had long-lasting effects for any future help-seeking attempts. Overwhelmingly, where participants had negative experiences, these stuck with the young people and affected their decision to seek help moving forwards. For some participants, this gradually built up with each consultation:

“*There was a growing hopelessness. There was a concern that I would get worse, become more distressed, more suicidal, maybe make an attempt.*”

(Mia)

Other participants had such negative experiences that they completely disregarded the GP as an option for any future help:

“*I think a GP is not an option anymore, to get help from them, in terms of my mental health. Because, it has happened with two separate GPs, I think it will happen again with another one*”

(Connor)

For each GP consultation that the young people found unhelpful, participants appeared to use this as a growing personal evidence base that they should not seek help from the GP again. If at this point, the young people did not have alternative access to support (such as a CPN), it was often the case that their suicidal risk would increase until they ended up in specialist services, bypassing the GP. This is particularly evident for the participants who also felt let down by the support that was offered.

“*That is the scary thing, nobody can do anything, apart from you being sectioned or whatever. This normally wouldn’t happen until you have tried to kill yourself. I feel like there is nothing that anyone can really do, other than give time to somebody, sit and listen to them and reassure them. But, this services barely exists to anyone who hasn’t already tried to kill themselves*”

(Olivia)

“*Each time I visit the GP, I would have a massive anxiety attack, because they are an authority figure and because they had been so cold and unhelpful.*”

(Mia)

“*I was expecting to be turned away. I was expecting to be shunned*”

(Sarah)

Olivia talks about how scary it can be thinking that there is nothing that can be offered unless you try and kill yourself. There is a sense that the support that is desperately needed is not available until you reach a crisis. Mia further highlights that previous bad experiences had a direct impact on her ability to attend future consultations, bringing on anxiety attacks at each visit. Finally, Sarah summarises this sense of growing hopelessness suggesting that her experience has led her to expect to be let down, ‘turned away’.

However, although it only took one negative experience in some cases to change the way a young person would seek help from a GP, other participants described how they were able to draw on positive experiences to give them hope. This again is particularly evident where young people knew they could see the same GP, and speak to someone who knew them. In the quote below, Olivia speaks about the contrast of seeing a familiar and unfamiliar GP when it comes to seeking help and support:

“*At times, I know that the only option to help me is through a crisis team. So there have been times where I have gone to my GP and said ‘look, can you call them and tell them what is happening’ and the GPs that do not know me would be like ‘why? You can ring them?’ and I can’t explain to them that I just can’t. The one that knows me though, she knows the drill, she knows my problems and she will do it.*”

(Olivia)

Sophia goes on to highlight how unpredictable the GP consultations can be. Many young people do not get to see the same GP, particularly if it is an emergency appointment. Even if they do see the same GP, there are many other factors at play that could influence the course and outcome of the consultation which makes the whole experience a “hit and miss” according to Sophia.

“*It is hit and miss. It depends on their personality and the day you catch them on, and everything. There are so many factors involved, but fortunately I have had good experiences.*”

(Sophia)

Young people understood and described the help seeking from a GP as an ever-changing and evolving process. What is apparent is how prior experience was one of the most important factors in determining whether the young person would seek help again in the future. For young people at risk of suicide, each interaction was significant and defining.

## 4. Discussion

In this study, we explored, through in-depth qualitative interviews, how, why and when young people seek help from a GP for suicide-related experiences. The findings, grounded in young people’s perspectives and experiences, offer an original contribution to our understanding of help seeking from primary care with significant implications for clinical practice. Help seeking was described by young people as a dynamic process that changes over time and is influenced by a range of different factors. *Prior* to the first GP consultation, there was an emphasis on how a young person comes to understand and articulates their distress, the importance of their informal support networks and their perception of the GP as a potential source of help. *During* a GP consultation, willingness to seek help was influenced by how safe and supported the young person felt to disclose their distress. Perceived GP training, verbal and non-verbal communication skills and validation of their concerns were highlighted as important factors to help facilitate this process. Subsequent help seeking was then largely influenced by prior experience of GP consultations and the availability of alternative support. The findings of this study also show that young people are aware of the negative attitudes that GPs might hold in relation to self-harm and suicidal behaviour. This awareness can make the process of help seeking an invalidating experience and, in some cases, the young people felt like it led to an exacerbation of self-harm or suicidal behaviours among the participants in our study. Where self-harm or suicidal behaviours did escalate some participants would present directly to the A&E as opposed to visiting their GP. Previous research suggests that this might be due to the time of presentation, for example, usually out of hours; and/or the severity of the injury which might require immediate medical attention [35,36,37]. However, the negative experiences of young people attending A&E have also been highlighted in the literature, with self-harm patients viewed more negatively than other patients by hospital staff [38,39]. Young people have spoken about the shame, discrimination and punitive treatment they received that led to a further cycle of self-harm and avoidance [36]. In the present study, attendance at A&E did not always lead to additional support and participants often still had to use their GP as a gatekeeper for additional support in the long term. It is unsurprising therefore that young people spoke about feeling hopeless about seeking help as the only available pathways into further mental health support have been shown to be, on some occasions, deeply invalidating experiences.

There have been a number of theoretical models explaining the process of help seeking among young people. Among the most dominant, is Rickwood and colleague’s [19] which suggests the process of help seeking starts when a young person becomes aware of their symptoms and decides that they have a problem that could benefit from intervention. The following stages of help seeking suggest that once a problem has been identified, the person then needs to be able to articulate or express their internal stress in a way that can be understood by others. Finally, for a young person to access help successfully, it is suggested that once they are able to articulate how they feel, the source of help must then be available and the help seeker must be willing to disclose their internal distress. In the present study, young people were not necessarily struggling to identify or acknowledge their distress or symptoms. However, they struggled to understand what could be done with their symptoms and it was not always clear to them as to who could help them and how. This may go some way to explaining why so many young people primarily prefer to seek help from their informal support network [20,40,41]. Furthermore, as Klineberg and colleagues had suggested, the informal support network also plays an important role in when and how the young person attended a GP consultation [22]. Participants suggested that the experience, knowledge and availability of their support network affected the process leading up to the first GP consultation.

In this study, young people often did not consider the GP as a source of help for their suicidal experiences and associated distress; they also believed that GPs were not sufficiently trained to help. Importantly, some of the participants were worried about the stigma of attending a consultation and being mislabelled as attention seeking. We know from previous research [2,37,42,43] that some of the most common misconceptions held by health professionals about self-harm and suicidal behaviours refer to these as being “attention seeking” or simply part and parcel of being “a troubled adolescent”. Findings from our study suggest that young people may be aware of negative attitudes and misconceptions that some GPs may hold, and had come to accept and internalise these, believing that if they sought help, then their experience would no longer be valid. In a recent study by Bellairs-Walsh and colleagues [44], young people spoke extensively about the impact that such misconceptions and negative labels could have on the disclosure of suicidal or self-harm behaviour; thus, highlighting, the significance of beliefs, attitudes and stigma on the early stages of help seeking [21,39]. Despite the negative attitudes towards seeking help through a GP, young people were aware that this is an essential process to receiving further help, and this itself is a barrier that has been highlighted in previous research [36]. The motivation to speak with a GP about suicide and self-harm was therefore seen as a necessity rather than a preference. In the present study, some young people, who have accessed secondary care on more than one occasion, spoke about learning what they need to say to be able to ‘jump through the hoops’ quicker to get the right support. On the other hand, those who were unable to do this, or had particularly negative experiences, were often put off from seeking any formal help in the future. Recent policy initiatives in the UK highlight the importance of integrating mental health professionals into primary care as a way of facilitating access to care and reducing the impact of mental health consultations on GP workload [45]. Important lessons could also be learned from innovative service delivery models (Headspace) in Australia and across the world facilitating integrated youth mental health care [46]. The findings of our study reinforce previous calls for the need to have enhanced and integrated primary and community mental health services as a way of providing a more coordinated and personalised care to young people at risk of suicide [37].

In line with other research [21,39], this study suggests that young people could not always find the right words to articulate their distress; and would instead refer to physical symptoms or metaphors to try and explain how they felt or rely on their informal support network to understand and articulate the distress on their behalf. Participants presented to their GP sometimes in silence or sometimes gave hints or clues to how they felt, hoping that the GP would help make sense of this experience and offer the right support. There was also a sense that the young person should not necessarily have to be able to articulate how they feel; it was the job of the GP to help with this process by asking direct questions and unpicking the experience. Taken together, the findings of this study highlight that young people did not always go through all of the proposed stages of help seeking outlined in previous models [19]. For example, in this research young people were able to access help at times even when they were unable to articulate how they were feeling. It is therefore possible that the stages of help seeking may not be as discrete as proposed [19] or there may be alternative pathways to accessing support that is not yet fully captured in existing models of help seeking [40].

Barriers and facilitating factors to help seeking from a GP have been highlighted as being important in understanding when, how and why people seek help. Gulliver and colleagues suggested that such barriers included poor mental health literacy, preference to be self-reliant, and concerns about stigma [39]. The present study further highlights the importance of GPs’ effective verbal and non-verbal communication and creating a space where the young person can feel safe and able to disclose risk. Indeed, Murray’s model of help seeking, highlights how help seeking can be influenced by the perception that something can be done about the problem [40]. However, in the present study, young people acknowledged that sometimes not much could be done to help, but being heard, validated and taken at face value were as important, if not more important, to their willingness to disclose how they felt. Participants spoke about being aware of long waiting times for interventions, time-limited consultations, and a sense that they could burden the GP with their distress. However, whilst this suggests that there are practical aspects of the GP consultation that limit availability, it also further highlights the significance of how young people interpret and make sense of the available help-seeking pathway.

The most important facilitating factor to future help seeking for young people was prior experience of a GP consultation. Many of the young people interviewed had negative experiences at GP consultations which made them reluctant to seek help again. For some, the experience was so negative that it actually exacerbated their self-harming or suicidal behaviour following consultation. Where an increase in suicidal behaviour was described, participants typically also described a growing sense of hopelessness. However, some participants also felt that they needed to escalate their behaviour to match their internal state before they would be taken seriously. For example, where a GP minimised the severity of a young person’s suicidal experience, there were examples described where the young person increased their self-harm, by cutting more ‘deeply’, to try and justify why they needed help and that their experience of distress was genuine. This is line with previous research [44], where young people reported feeling compelled to escalate their behaviour or amplify the extent of their distress in order to be taken seriously or convince their GP about the need for referral to specialist services.

### 4.1. Strengths and Limitations

This is the first study to offer an in-depth exploration of the processes involved in seeking help from a GP when young people have felt suicidal. There has been little exploration of help seeking from primary care amongst suicidal young people despite the fact that GPs are often the first and last health care contact for those who die by suicide [43]. A strength of this study is the use of a framework analysis approach allowed for a rigorous and clear research audit trail for other researchers to follow. The use of the second researcher for cross validation enhances the credibility of the findings. This study was presented in line with the Consolidated Criteria for Reporting Qualitative Research (COREQ) ensuring the explicit and comprehensive reporting of all study elements. This study also has some limitations that need to be acknowledged. The sample size of eight participants, although allowed for detailed exploration of each individual account, is considered small. In addition, convenience sampling based on accessibility, interest in this study and willingness to participate might have led to over-representation or under-representation of particular groups of young people within the sample. Participants self-selected to take part and therefore there is a likelihood of selection bias in the sample. It is therefore possible that participants in this study are not representative of young people with suicidal tendencies. Nonetheless, the study findings reflect the richness in young people’s experiences of help seeking from a GP which included both positive examples of practice but also areas for improvement.

### 4.2. Implications for Research and Clinical Practice

Our study highlights that help seeking for suicidal experiences is a dynamic, non-linear process influenced by different factors. Future theoretical models of help seeking for young people need to focus more on conceptualising and capturing the dynamic and fluctuating processes, such as past experiences of help seeking, available support networks and young people’s ability to articulate internal distress. For example, in the present study, young people who have been discharged from secondary care back to their GP changed their approach to help seeking based on their past experience. Participants who had positive experiences or good relationships with their GP found it easier to disclose and seek help for their suicidal ideation. However, those with poor experiences of care either learned how to ‘jump through the hoops’ to get back to secondary care or they relied more heavily on their support networks.

There is an urgent need for future studies to explore how existing models of help seeking apply in particular contexts and cultures, as it is likely that the process of seeking help will vary based on the presentation, support pathway cultural context and socio-economic determinants. For example, previous studies have shown that young South Asian women seek help from primary care only when in crisis and as a last resort [47]. As their experiences of care following a self-harm episode are usually negative, they are often hesitant and apprehensive to seek help in the future [47]. It is important therefore for future research to explore how help seeking (formal and informal) is understood and conceptualised by individuals from different ethnic and cultural backgrounds [48], and develop and evaluate models of help seeking which are culturally sensitive.

The findings of this study have important implications for specialist training and education on youth suicide prevention in primary care. This, in line with previous research [37], suggests that training should extend beyond the provision of micro-skills to a more holistic and integrated approach to understanding youth mental health, including self-harm and suicide risk. In line with the National Institute for Health and Care Excellence (NICE) recommendations [49], a psychosocial assessment of risks and needs can facilitate therapeutic engagement and communication with young people who self-harm. In addition to this, we propose specific training for GPs to enhance clinical skills in eliciting and responding to suicidal ideation, asking sensitive questions and communicating with young people around topics which could be considered sensitive and potentially distressing [36]. Non-verbal communication signs such as eye contact, facial expressions, posture and tone of voice were among those elements perceived by young people to demonstrate empathy and warmth on behalf of GPs during consultations. Compassion and validation of young people’s concerns are important aspects of building rapport and a therapeutic relationship which in turn could facilitate future help seeking. As such, the assessment of suicide risk should not be seen as a discrete process; rather, as an ongoing process that may take place over a number of consultations and might require the involvement of other sources to fully understand a young person’s suicidal risk and associated distress. In addition, it is perhaps preferable to young people that in the long term a system that integrates mental health support within primary care may be a better option to facilitate help seeking [46,50,51].

The present study shows that young people may not always be able to find the right words to explain how they are feeling. Previous research highlights that young people often present to primary care with somatic complaints, such as headaches and stomach pains, which could reflect the difficulty many of them experience when it comes to articulating their distress or mental health concerns [52]. Being mindful of such less overt presentations is important for clinical practice as it offers an opportunity for early identification and timely management.

Our findings further highlight the importance of involving family and/or friends in primary care consultations with young people. Young people value the presence of an informal advocate particularly when the consultation involves discussing potentially sensitive or distressing topics where some young people might simply not have the right words to explain or articulate how they feel and why. Whilst family/friend involvement in consultations is important, confidentiality is something a lot of young people are concerned about [44]. GPs should ensure that they are discussing with young people how they wish information to be shared, and with whom. Wherever possible, this should also happen if there is a serious concern over suicide risk. It is important for any professional to offer time with and without the young person’s friends and family to allow for the young person to disclose information that they may not want their parents to hear. Importantly, this recommendation is also in line with the information sharing and suicide prevention consensus statement [51].

## 5. Conclusions

This study is grounded in young people’s perspectives and experiences in order to provide an in-depth understanding of help seeking at a time that they have felt suicidal. This study highlights that help seeking from primary care is a complex process for young people, which does not appear to follow the discrete and sequential stages proposed in existing theoretical models. The findings of this study offer new insights and recommendations to inform theoretical models of help seeking, future research and clinical practice and provision of specialist training and education. Importantly, the holistic and individual needs of young people need to be taken into account at each consultation and reassessed across each episode of help seeking. This would include their past experiences, ability to articulate distress and the availability of their support network. The negative experiences that young people in our study reported demonstrate that previous recommendations are yet to be fully addressed in clinical practice and this remains a significant barrier to young people at a time when they are most vulnerable. 

## Data Availability

The data presented in this study are available on request from the corresponding author. The data are not publicly available to protect the anonymity of the participants.

## Supplementary Materials

The following are available online at https://www.mdpi.com/1660-4601/18/4/2120/s1, Figure S1: Example of coding; Figure S2: The Framework Matrix; Figure S3: Example of charting the data into the matrix; Figure S4: Example of how the data was summarised at the start of the interpretative stage.

## Appendix A

Figure Appendix A: Semi-Structured Interview

My name is XXX.

Thank you for agreeing to participate.

Purpose:

- Who we are, and what we are trying to do
- What we will do with the information
- Why you were asked to participate

Interview Questions:

I understand that about X [insert number] months/years ago, things in your life got a bit too much and you found yourself at a very vulnerable place. This must have been very hard and stressful both for yourself and your family. I’d like to ask you a few questions about the services and support you received during that difficult period in your life.

1. Can you tell me briefly about your experience of attempting to end your life?
  Prompts: what was happening at this time? How did you understand what was happening for you at this time? What sense did you make of this?
2. Who did you first contact to seek help and support?
  Prompts: If GP, what was the reason you chose your GP to talk to first?
  If not the GP, did you consider contacting your GP at that point?
3. If you found yourself in the same position, who and why would be the first person you would contact to ask for help?
  prompts: what is your reasoning for this? Why do you think this is? Do you have any other coping strategies? What was your support network like before/ now? Isolation? Physical Health?
4. Why did you choose to seek support at this time?
  prompts: was there anything you noticed about yourself or how you were feeling? Was anybody else involved in suggesting you should access support?
5. How did you find your experience visiting your GP?
  Prompts: Did you go by yourself or did someone come along with you? If someone came along was that helpful?
6. Did you have any hopes or concerns before visiting your GP?
  prompts: did these hopes or concerns play a role in deciding whether you would seek support? What did you think the outcome would be?
7. What services were offered to you?
  Prompts: how happy/unhappy were you with what was offered?
8. Do you think that GPs are the first point of contact for mental health issues? If not, who do you think is?
  Prompts: Did this play a part in deciding whether to talk to your GP about your difficulties? What do you think the GPs role is in regards to mental health difficulties
9. Could you talk me through the process you went through from your suicide attempt to seeking support, what was important to you?
  Prompts: Could you highlight anything that felt significant to you?

Thank you for taking part in this interview. Is there anything else you can think of that we have not covered?
