# Supplementary material for: Exploring the Processes Involved in Seeking Help from a General Practitioner for Young People Who Have Been at Risk of Suicide
Source: Int J Environ Res Public Health. 2021 Feb 22;18(4):2120. doi: 10.3390/ijerph18042120 (PMC7926569; doi:10.3390/ijerph18042120)
Supplement: Supplementary file 1 [file ijerph-18-02120-s001.pdf]

# Example of coding

*Example from Transcript 1 (Prisha):*

| | | |
|---|---|---|
| 538: importance of GP's providing information about other services<br><br>541: Normalisation would have been helpful | **Prisha**: I think, I had a really good experience. I think the one thing that could have been slightly better, because it was handled almost perfectly, I think the one thing that I think I would have gained from it, was some more information about what CAMHS meant, and what kind of help I could have got and if I could, If I was told that there are other people that go through things like that, and that I wasn't alone. Maybe, having information about support | 538: It would have been helpful to have more information about services such as CAMHS<br><br>541: would have valued normalising her experience. That other people feel this way |

*Example from Transcript 2 (Connor):*

| | | |
|---|---|---|
| 350: GP consultation viewed as unhelpful<br><br>351: Suicidal thoughts increased following consultation<br><br>355: Found the way the GP communicated made him more suicidal | **Connor:** From the two male GPs really unhappy because I just felt I was just being left to wait to see a psychiatrist. Like I said, if the CPN hadn't have stepped in then I definitely would've made an attempt on my life. It would have been their fault because it wouldn't have been my decision to do that. They were the ones that should have not even-- Okay, I understand about not giving me medicine because I'm complex, but the things that he said like other people are worse off, he didn't have to say that at least. | 350: Felt let down by the GP, just told to wait.<br><br>351: Blaming of the GP for the increasing suicidal thoughts because they did not step in<br><br>355: Understanding that GPs are limited in what they can offer, but how they communicate with Connor was important |

## The Framework matrix

| Theme / Subtheme | Description |
|---|---|
| **1.Young person's early experiences** | |
| 1.1 Development of suicidal thoughts / behaviour | Description of how the young persons suicidal thoughts or behaviour developed. This could include the age this started, what they noticed, what method(s) were used or the frequency of these behaviours or feelings. |
| 1.2 Description of support network | Description of the young persons support network at the time their suicidal thoughts or behaviour was developing. This could include their friends or family and a description of what the support might have looked like. |
| 1.3 Adverse childhood experiences | This is a description of any adverse childhood experiences that may have occurred. This could include difficulties at home or school and covers different types of abuse and trauma the young person may have been subjected to. |
| **2. Understanding of young person's difficulties** | |
| 2.1. Understanding of internal experience | This is how the young person made sense of their difficulties, their understanding of mental health difficulties and the language used to explain their internal state. |
| 2.2 Understanding within young person's support network | This is how other people around the young person made sense of the young person's difficulties. This could include significant others such as family, friends or school staff. |
| **3. Seeking support from a GP** | |
| 3.1 Young person's view of GP role | This is the perception the young person has of the GP, what they could offer, their hopes and concerns. |
| 3.2 Process leading up to GP consultation | This is the process that led up to the first GP consultation. Did they go directly to the GP? Did they go alone, or with someone? Was it the first point of contact, or were other forms of support used first? |

| | |
|---|---|
| 3.3 Drawing on past experiences of GP consultations | This describes how young people draw on past experiences of GP consultations to inform future help-seeking choices. |
| 3.4 Presenting at other medical settings | This is a description of where the young person came into contact with services if the GP was not their first contact. Examples include A&E or Psychiatric inpatient units. |
| **4. Description of GP consultation** | |
| 4.1 What support was offered | This is a description of what was offered following the GP consultation. For example, were they referred on for further support? Did they receive medication? |
| 4.2 The young person's experience of the GP consultation | This is the young person's personal experience of the GP consultation, how they felt during the consultation and what they took from the visit. |
| 4.3 Facilitating factors at the GP consultation | This is the young person's view of what factors helped at the GP consultation. This could include anything that helped them feel heard, understood and able to communicate their difficulties. |
| 4.4 Barriers at the GP consultation | This is the young person's view of what factors may have made the GP consultation less helpful. This could include anything that prevented them to feel heard, understood or able to communicate their difficulties. |
| **5. Post GP experience** | |
| 5.1 New sources of support | This is a description of how the young person's source of support may have changed since their GP consultation. This could mean that if relapse happens they now have a support network. |
| 5.2 Reasons for relapse | The reasons provided why a young person relapsed or required further help from mental health services. |

# Example of charting data into the matrix

| Theme | 2. Understanding of Young Person's Difficulties | |
|---|---|---|
| **Sub Theme** | **2.1 Understanding of Internal experience** | **2.2 Understanding within young person's support network** |
| Prisha | Suicidal experience linked to other diagnoses (ASD, Attachment difficulties) (Line 51-60) | Parents did not understand Prisha's difficulties (Line 58-60) |
| | Found it hard to understand internal feelings (118 - 120) | Parents did not know what to do with the self harm (Line 204-208) |
| | | School was too big to be able to notice warning signs for students (342-345) |
| Connor | Sad all the time, anxious - didn't know why (23-24) | Mum noticed Connor was sad / depressed (24) |
| | Little things change, stop eating, not sleeping, crying all the time, getting worse (223-226) | Mum suggested the GP would help make sense of what was happening (255-257) |
| | Didn't know what depression was (253-254) | |
| | Depression was initialy light and easy, then became dark and heavy (285-287) | |
| Hayley | Couldn't understand what I was feeling, didn't know depression existed (QQ? 22-30) (56-58 - Q) | Frinds noticed Hayley was missing, and were worried - looked for help (114-119) |
| | Didn't make sense - Depression was selfish, she felt bad, she needs help - didn't consider others (26-52 | Different friendship groups notcied suicidality before her (111)(114-119) |
| | Only with experience was she able to realsie the feelings she had were 'suicidal' (105-107) | Teachers noticed her mental health declining (everyone noticed before her) (241-248) |
| | Usually expressive, if I notice I am quiet I think there might be something wrong (214-216) | Frineds thinking 'what the fuck? Is she on crack?' (389-395) The fuck is happening (417) |
| | notices physical symptoms of bi-polar, signs things might be going wrong (220-227) | |
| | Felt like normal sleeplessness, typical uni behaviour, but then started doing weird shit (369-377) | |
| | Friend used the word manic - thought whats that? Not heard that before? (401-412) Q? | |
| | People don't have insight, so don't seek help (585-592) | |
| | Noticed phsyical symptoms - realised the body says what the mind cant (750-754) | |
| Sarah | I didn't understand what was going on, I was an overflowing cup (37-42 QQQ) | Friends and family (pretty much everyone around her) noticed before her (252-256) |
| | A depth to my struggles, I didn't have the words to explain (146-14* QQQ) | Other people understood her difficulties, eyeopening experience for Sarah (659-663 QQQ) |
| | I thoguht I was doing fine, other people were the ones who noticed (252-256) | |
| Mia | good insight - difficulties built up and led to suicidal feelings (77-79) | Parents were very concerned, they called home treatment. Couldn't be around to look after her (24-37) |
| Sophia | Couldn't come to terms with what was going on in her life, wanted a way out (51-59) | Mum said part of her behaviour were abnormal (359-361) |
| | Link with diagnosis of anorexia (54) | Family never had any difficulties with MH, mum was scared, didn't know what to do (523-532) |
| | Saw first attempt as significant, didn't know what to make of it, until in hospital (60-66) | Mum pointed out the behavioural pattersn she recognised as OCD (668-678) |
| | I didn't know what the hell was going on, screaming inside, but didn't want help (456-458 QQ) | Mum heard about the self-harm became concenred, had to go to a DR (699-709) |
| | Part of me hoped nobody noticed, I want it be really bad to be disgusting (486-488 QQ) | Mum also questioned eating disorder (717-718) Dad said self harm was normal (702) |
| | Never heard of OCD, it was scary when it was pointed out (668-678) | |
| | After self-harm what have I done? (690) Then became addicted to it (707-709) | |
| | Why are you asking me if I have an eating disorder? I was too big to have an eating discorder (710-719) | |
| Olivia | Detatach from the experience (33-34) | Friend also was a bit more clued up on mental health, said social anxiety (198) |
| | It is a private experience, didn't want help (41-44) I tolerate it, up until I cant (53-57) | Classmate in school told parent, parent told school, everyone was angry. Made Olivia want to cope alone (344-354) |
| | I had horrific anxiety, but didn't know what it was, didn't understand mental health as a concept (186-193) | |
| | Only after a diagnosis of ASD did things start to make sense (284-296) | |
| | experience started to get worse when I identified with other people and their stories (567-578) | |
| Liam | Understood expereinces as fight or flight - anger gets most of him - not nice having to watch your back a | Dad understood his difficulties because older sister also had mental health difficulties (98-100) |
| | Withdrew and began playing games all the time, spending all his time in his room (86-89) | Family isnt open about mental health difficulties, don't show understanding (121-130) |
| | "I didn't understand my mental health growing up" hard to talk about (95-96 Q) | Once Liam contacted sisiter, sister said she could see him struggling for a while (169) |
| | The stress built up over time, until it was just on top of him (163-164) | Dad has foster kids, and has supported them so knew the processes (227-233) |
| | I was confused myself, it was hard to say how I was feeling (177-178 QQ) | Nobody actually understood (239-240) |
| | Only understood internal state when in hospital with other people (252-254) | |

## Example of how the data was summarised at the start of the interpretative stage

### 1.1 Understanding of internal experience

There was a general theme across the participants around having difficulties understanding and making sense of their internal feelings. Many of the participants were aware that something was not right, but they talked about having a lack of understanding around mental health difficulties and this made it more difficult to talk about and seek help for their difficulties.

**To me, it seems it was almost like an overflowing cup. A cup, which kept getting full, filled up, and then there was just one night and – Again, I can't say what happened, it was too much. (Sarah, Page 2, Line 38)**

Some participants tried to use the physical symptoms to be able to make sense of what they were feeling.

**I just had this horrific anxiety, but I didn't know what it was. I didn't really understand mental health as a concept. Then I described to my friends that before we were going to go out or before we were going to do something, I just had this feeling of being sick or not being able to go. (Olivia, Page 7, Line 191)**

One participant summarised this experience by saying:

**The body tells you what the mind can't (Hayley, Page 27, Line 751)**

Each participant used different language and ways of describing their experience; the commonality was that at the time they didn't feel they had the right words or language to explain their experience.

**When it first started, it was, this sounds bad, but it was easy and it wasn't intense. It was light, if that makes sense. But when it came to trying to end my life, it was so dark and so heavy. (Connor, Page 10, Line 285)**

**I think there is such a depth to my struggles that I didn't quite have the word to explain. I didn't want to be turned away because I was explaining it in a lighthearted manner (Sarah, Page 6, Line 147)**

Some participants used diagnoses to help make sense of their experience (such as ASD, Anorexia or OCD). Others talked about only understanding their difficulties when they were older and had gone through services, and some talked about truly understanding when they had met others who were experiencing similar difficulties.

**I didn't really understand until I got put in hospital around patients with mental health difficulties, that I really, truly, understood what was going on (Liam, Page 9, Line 252)**
